# Utility of novel echocardiographic measurements to improve prenatal diagnosis of coarctation of the aorta

**DOI:** 10.1038/s41598-023-31749-8

**Published:** 2023-03-25

**Authors:** Takuya Fujisaki, Yoichiro Ishii, Kunihiko Takahashi, Masayoshi Mori, Kumiyo Matsuo, Dai Asada, Hisaaki Aoki, Sanae Tsumura, Shigemitsu Iwai, Futoshi Kayatani

**Affiliations:** 1 Department of Pediatric Cardiology, Osaka Women’s and Children’s Hospital, 840 Murodo-Cho, Izumi, Osaka 594-1101 Japan; 2 Department of Cardiovascular Surgery, Osaka Women’s and Children’s Hospital, Osaka, Japan

**Keywords:** Cardiology, Medical research

## Abstract

Prenatal recognition of coarctation of the aorta (CoA) may improve neonatal survival and reduce morbidity. However, prenatal diagnosis of CoA remains challenging, with relatively high false-positive and false-negative rates. This study aimed to identify a novel formula based on fetal echocardiographic measures to predict prenatal identification of CoA. A retrospective comparison on the echocardiographic evaluation of 30 patients with suspected CoA between May 2016 and April 2021 was performed. The patients were divided into a postnatal surgical intervention group (n = 13) and a non-intervention group (n = 17). The measurements that showed significant differences were aortic isthmus diameter Z-score (p < 0.001), ductus arteriosus diameter/aortic isthmus diameter (p < 0.001), and distal aortic arch (DA) index (p < 0.001). In the receiver operating characteristic curves analysis, the DA index was the largest with an area under the curve of 0.941 and a cutoff value of 1.28, with a sensitivity of 85% and a specificity of 94%. Measurement of the DA index improved the diagnostic rate of fetal CoA and a DA index ≧ 1.28 indicated fetal CoA cases requiring surgical intervention.

## Introduction

Coarctation of the aorta (CoA) is one of the most common congenital heart defects in the pediatric population, accounting for 4–8% of all infants with congenital heart defects^1, 2^. CoA is defined as a discrete narrowing of the aorta in the region of the ligamentum arteriosum and distal portion to the origin of the left subclavian artery, although more diffuse forms of the disease may involve the arch or isthmus to varying degrees^3^. If CoA is not diagnosed at the prenatal stage or in the immediate neonatal period, aortic obstruction with distal hypoperfusion, metabolic acidosis, renal injury, left ventricular (LV) dysfunction, pulmonary edema, and pulmonary hypertension may occur^4, 5^. Prenatal detection of CoA remains a challenge despite its importance, which is indicated by the significantly higher mortality and morbidity in newborns without prenatal detection^4, 6–8^. Several studies have described how detect CoA prenatally, but all have multiple measurement sites and complicated formulas. In addition, false-positive and false-negative rates are reportedly high^9^. Recently, the ratio of the aortic arch diameter at the left subclavian artery to the distance between the left carotid artery and the left subclavian artery has been described as being significantly longer in neonates and infants with CoA^10^. Therefore, we considered the possibility of applying this test to the diagnosis of CoA during the fetal period. We aimed to investigate a new criterion to improve the diagnostic rate of aortic constriction during the fetal period.

## Methods

### Patients and methods

**Figure 1:**
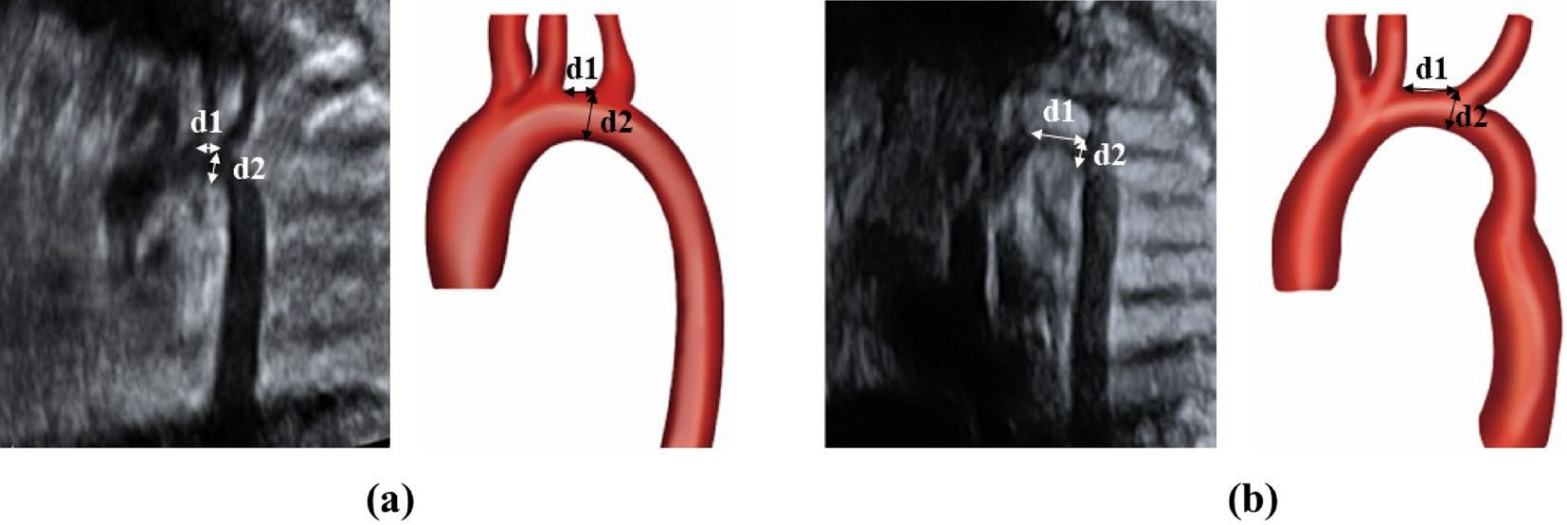
Echocardiographic and enhanced computed tomography scheme of an aortic arch (DA index = d1/d2). Echocardiographic and enhanced computed tomography image of a normal aortic arch with normal DA index (**a**) and CoA with abnormal DA index (**b**). The DA index was the ratio of the distance between the origin of the left carotid artery and the origin of the left subclavian artery (d1) to the diameter of the distal aortic arch at the origin of the left subclavian artery (d2). *DA* distal aortic arch, *CoA* coarctation of the aorta.

All the patients provided written informed consent. All the procedures were performed in accordance with the principles of the Declaration of Helsinki. The study protocol was approved by the Institutional Ethics Review Board of Osaka Women’s and Children’s Hospital (No. 1354). This retrospective review assessed 30 patients with suspected CoA between May 2016 and April 2021 at the Osaka Women’s and Children’s Hospital. We excluded cases with complicated congenital heart diseases such as single ventricular disease and transposition of great arteries. CoA was diagnosed during pregnancy at approximately 30 weeks of gestation. Maternal, prenatal, and postnatal medical records, including cardiac surgery reports, were reviewed for gestational age, chromosomal abnormalities, echocardiographic findings, and fetal/neonatal clinical courses. Echocardiographic studies were performed using the GE Voluson E10 equipment. A total of 10 cardiovascular dimensions were calculated from fetal studies around 30 weeks of gestation. General heart measurements included cardiothoracic area ratio and total cardiac dimension. Right heart measurements included the right ventricular (RV) end-diastolic dimension, tricuspid valve (TV) diameter, pulmonary valve (PV) diameter, main pulmonary artery diameter, and ductus arteriosus diameter. Left heart measurements included the left ventricular (LV) end-diastolic dimension, mitral valve (MV) diameter, aortic valve (AV) diameter, ascending aortic (AAO) diameter, ductus arteriosus, and aortic isthmus diameter. Ratios of the RV end-diastolic dimension to LV end-diastolic dimension (RV/LV), PV diameter to AV diameter, TV diameter to MV diameter, ductus arteriosus to aortic isthmus diameter (D/I), and the distal aortic arch (DA) index in the prenatal period have been calculated, and have been indicated as simple and noninvasive parameters to detect CoA in neonates and infants^10^. In this study, the DA index, which was calculated as the ratio of the distance between the left carotid artery and the left subclavian artery, was used to screen for patients with CoA (Fig. 1).

According to the clinical course after birth, patients were divided into two groups: the postnatal surgical intervention group (group S), and the non-intervention group (group N). Prostaglandin E1 continuous infusion after birth was administered based on the last two prenatal assessments and following the initial postanal echocardiogram. Confirmation of definitive CoA and decision to perform neonatal surgical repair were made based on clinical features of progressive heart failure and/or echocardiographic features of flow acceleration or loss of pulsatility at the site of the aortic isthmus. In addition, surgical repair was decided based on contrast computed tomography findings, including narrow isthmus morphology with a posterior shelf and/or hypoplastic arch morphology. If CoA was suspected based on echocardiographic estimation, the aortic arch was closely observed until ductal closure. All the clinical, echocardiographic, and operative data were reviewed. Follow-up in the neonatal period was completed for all 30 patients. Fetal cardiac dimension measurements were compared between the two groups.

### Statistical analysis

Data are shown as numbers, millimeters, Z-score, range, and mean ± standard deviation. Continuous and normally distributed data were analyzed using the Mann–Whitney U test. Categorical variables were analyzed using Fisher’s exact test. The area under the curve (AUC) and 95% confidence interval of the receiver operator characteristic (ROC) curve were computed using the predicted probability of the diagnosis of CoA. All statistical analyses were performed using EZR (Saitama Medical Center, Jichi Medical University, Saitama, Japan), a graphical user interface for R (The R Foundation for Statistical Computing, Vienna, Austria). It is a modified version of the R commander designed to add statistical functions frequently used in biostatistics. A *p-*value < 0.05 was considered to be statistically significant. All morphologic parameters and distance measurements were made by two independent observers (T. F. and Y.I.), who were blinded to the final diagnosis. There was no significant inter-observer variability in the echocardiographic measurements.

## Results

### Patient demographics

A total of 30 pregnant women and their fetuses (11 boys and 19 girls) underwent echocardiographic examination due to suspicion of CoA and referral from obstetrics and needed postnatal management, and underwent surgical repair, if needed, based on postnatal diagnosis. A total of 13 patients required surgical repair of the aortic isthmus portion (S group) and 17 patients did not need postnatal surgical repair (group N). The patient characteristics are shown in Table 1. The mean gestational age based on the evaluated fetal echocardiogram was 31.6 ± 2.0 weeks (range 28–35 weeks). The mean gestational age and body weight at birth were 38.5 ± 2.4 weeks (range 32–41 weeks) and 2732 ± 598 g (range 1140–3566 g), respectively.

**Table 1:** Patient and clinical characteristics of each group patients (n = 30).

	Group S (n = 13)	Group N (n = 17)	p-value
GA at echocardiography (weeks)	31.4 ± 2.0	31.8 ± 2.0	0.702
GA at birth (weeks)	38.2 ± 2.1	38.6 ± 2.7	0.325
Birth weight (g)	2684 ± 561	2769 ± 639	0.592
Male, n (%)	5 (38%)	6(35%)	1
Ductal shock	0	0	–
Cardiac complication, n (%)			
VSD	10 (77%)	4 (24%)	0.009*
PLSVC	3 (23%)	4 (24%)	1
BAV	3 (23%)	2 (12%)	0.628
Other complications	Kabuki make-up syndrome	Multiple abnormality syndrome	
	Heterotaxy syndrome	22q11.2 deletion syndrome	
	Turner syndrome	Heterotaxy syndrome Trisomy 21	

*GA* gestational age, *VSD* ventricular septal defect, *PLSVC* persistent left superior vena cava,; *BAV* bicuspid aortic valve.

*p < 0.05 between group S and group N.

### Clinical diagnosis and characteristics

For the patients in group S, the condition was identified by clinical manifestation and observed findings including narrowing of the aorta and the presence of a posterior shelf in the wall of the aorta, as well as other abnormal findings by Doppler imaging. There were no significant differences between the two groups in terms of the examination week, birth week, birth weight, or sex. Regarding intracardiac complications, 14 had ventricular septal defects (VSD) (46.7%), 7 had persistent left superior vena cava (PLSVC) (23.3%), and 5 had bicuspid aortic valve (BAV) (16.7%). There were significant differences in the presence of VSD, but no significant differences between PLSVC and BAV. In addition, genetic or chromosomal syndrome was diagnosed in seven neonates (23.3%), including 22q11.2 deletion syndrome, multiple malformation syndrome (auricle, external genitalia, and cardiac anomaly), Kabuki make-up syndrome, Turner syndrome, trisomy 21, or heterotaxy syndrome. There were no significant differences in the presence or absence of these syndromes. All patients were alive during this investigation and were followed-up after birth.

### Analysis of fetal echocardiographic parameters

**Figure 2:**
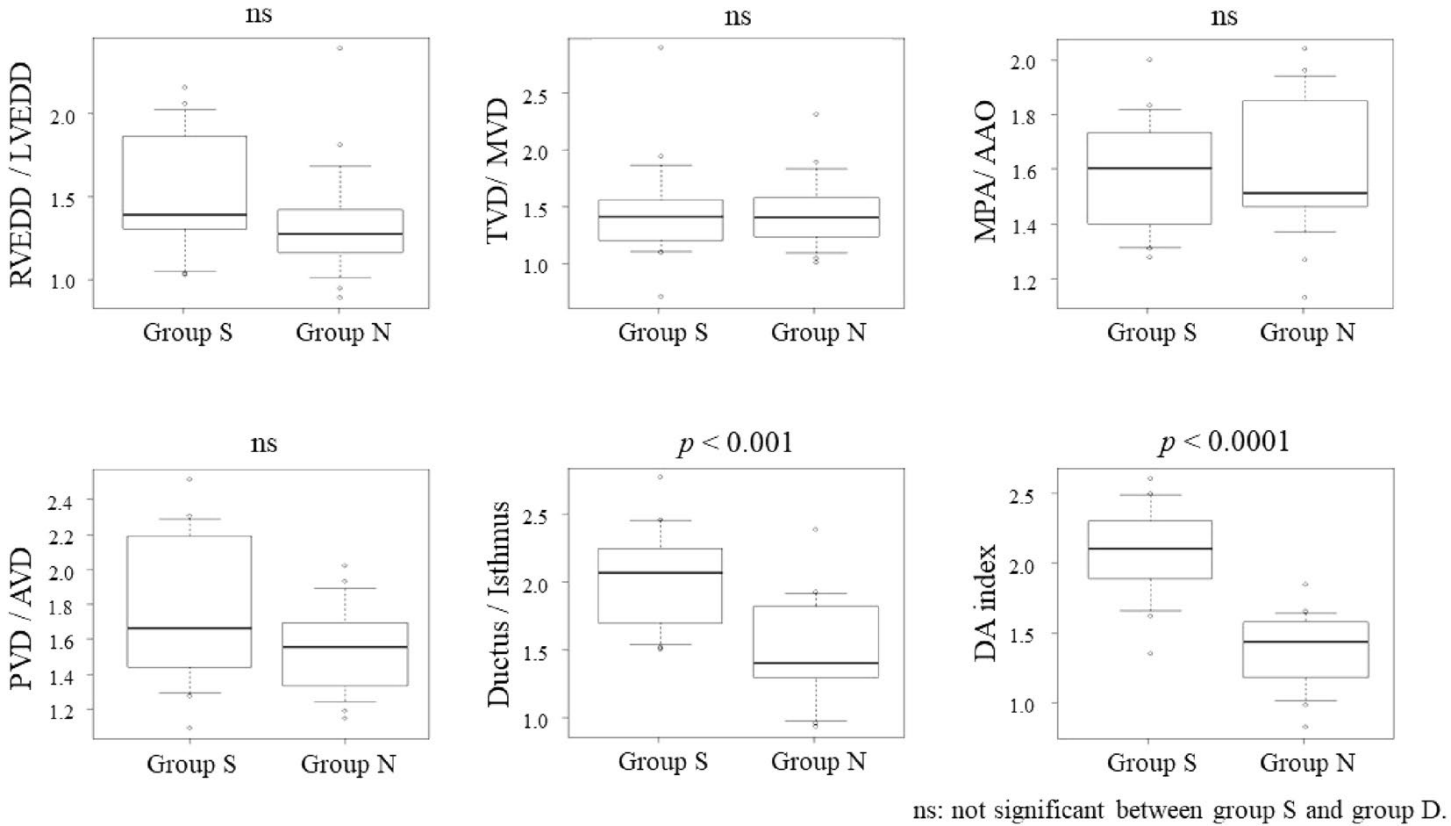
Ratio of cardiac parameter. Box-and-whisker diagram comparing different ratios of cardiac parameters. *RVEDD* right ventricle end-diastolic diameter, *LV* left ventricle end-diastolic diameter, *TVD* tricuspid valve diameter, *MVD* mitral valve diameter, *MPA* main pulmonary artery, *AAO* ascending aorta, *PV* pulmonary valve, *AV* aortic valve, *DA* distal aortic arch index, *ns* not significant.

Table 2 presents the Z-score of the fetal cardiac dimensions divided by the patient group. No significant differences were observed among the groups except for the Z-score of the aortic isthmus (*p* < 0.01). Table 3 and Fig. 2 present the ratios of the cardiac parameters for each group. The D/I ratio and DA index were significantly different between groups S and N (*p* < 0.001 and *p* < 0.0001, respectively). The inter-observer variability is shown in Supplementary Fig. S1.

**Table 2:** Fetal echocardiographic dimensions Z-score compared between groups.

	Group S (n = 13)	Group N (n = 17)	p-value
RVEDD	0.5 ± 1.2	0 ± 1.7	0.263
TVD	0.6 ± 1.6	0.9 ± 1.5	0.509
PVD	1.3 ± 1.6	1.0 ± 1.6	0.834
MPA	1.6 ± 1.3	1.5 ± 1.6	0.773
LVEDD	− 1.6 ± 1.0	− 1.3 ± 1.2	0.709
MVD	− 1.1 ± 1.8	− 0.9 ± 1.9	0.77
AVD	− 1.5 ± 2.3	− 0.9 ± 1.8	0.408
AAO	− 1.1 ± 1.5	− 1.3 ± 1.0	0.615
Ductus arteriosus	2.8 ± 2.3	2.8 ± 2.0	0.95
Isthmus	− 3.6 ± 1.0	− 1.3 ± 1.8	< 0.01*

*RVEDD* right ventricular end-diastolic diameter, *LVEDD* left ventricular end-diastolic diameter, *TVD* tricuspid valve diameter, *MVD* mitral valve diameter, *MPA* main pulmonary artery, *AAO* ascending aorta, *PVD* pulmonary valve diameter, *AVD* aortic valve diameter, *DA index* distal aortic arch index.

*p < 0.05 between group S and group N.

**Table 3:** Ratio of cardiac parameters and multiplier compared between groups.

	Group S (n = 13)	Group N (n = 17)	p-value
RVEDD/LVEDD	1.5 ± 0.4	1.3 ± 0.4	0.183
TVD/MVD	1.5 ± 0.5	1.5 ± 0.3	0.818
MPA/AAO	1.6 ± 0.2	1.6 ± 0.3	0.869
PVD/AVD	1.8 ± 0.4	1.5 ± 0.3	0.187
Ductus/isthmus	2.0 ± 0.4	1.5 ± 0.4	< 0.001*
DA index	1.6 ± 0.4	0.8 ± 0.3	< 0.0001*

*RVEDD* right ventricular end-diastolic diameter, *LVEDD* left ventricular end-diastolic diameter, *TVD* tricuspid valve diameter, *MVD* mitral valve diameter, *MPA* main pulmonary artery, *AAO* ascending aorta, *PVD* pulmonary valve diameter, *AVD* aortic valve diameter, *DA index* distal aortic arch index.

*p < 0.05 between group S and group N.

### Analysis of ROC curve

**Figure 3:**
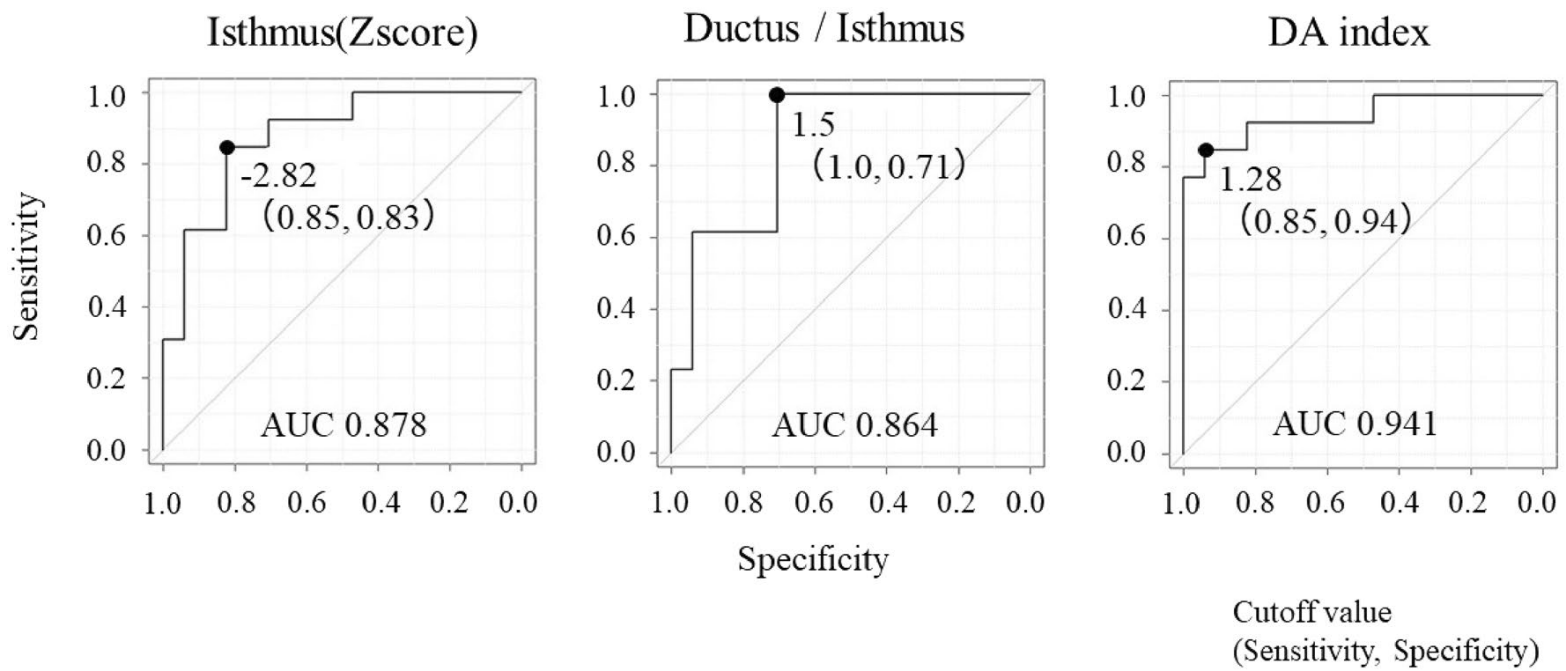
ROCs of three parameters with significant differences. ROC using the DA index had a sensitivity of 85% and specificity of 94% (AUC = 0.941). The optimal and most effective cutoff value is 1.28 when using the DA index to predict coarctation of the aorta fetuses. *ROC* receiver operating characteristic curves, *DA* distal aortic arch, *AUC* area under the curve.

The ROC curves for the three parameters with significant differences are presented in Fig. 3. The AUC for the DA index was found to be greater than that for the other two parameters. The DA index had a sensitivity of 85% and specificity of 94% with a cutoff value of 1.28.

### Follow-up

**Figure 4:**
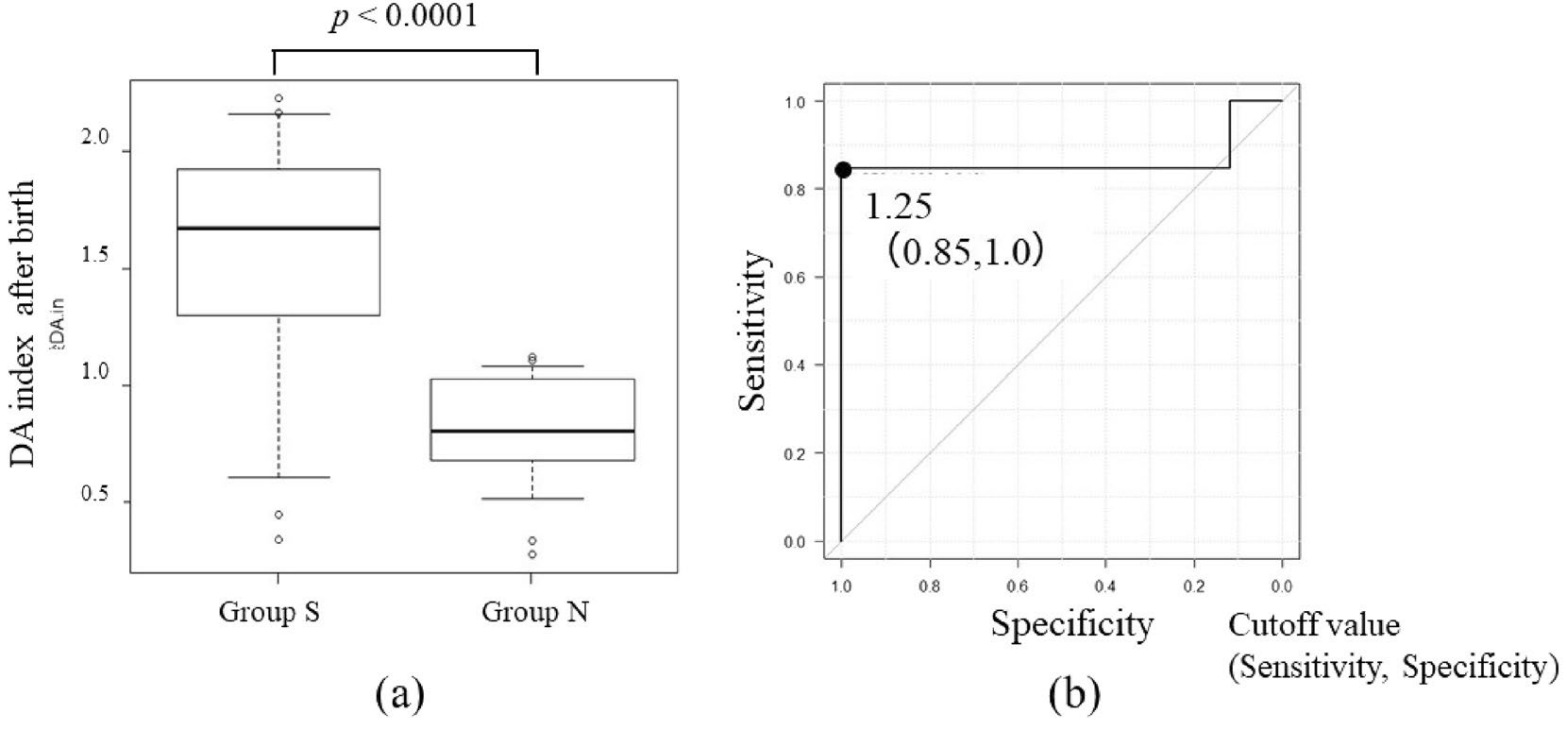
Ratio of cardiac parameter and ROC curve of DA index after birth. (**a**) Box-and-whisker diagram comparing each ratio of DA index after birth. (**b**) ROC of DA index after birth. *ROC* receiver operator characteristic curves, *DA* distal aortic arch.

All 13 patients in group S received postnatal PGE1 and underwent initial surgery on postnatal day 14.0 ± 10.9. Arch repair was performed with/without VSD closure in 11 patients and bilateral pulmonary artery banding in two patients. Only one patient with suspected CoA received PGE1 infusion immediately after birth. After stopping PGE1 infusion, patent ductus arteriosus closure was observed without circulatory collapse or need for surgical repair. The 17 patients in group N had an almost normal aorta confirmed by transthoracic echocardiography performed after the ductus arteriosus closed during a 12-month follow-up with no clinical evidence of aortic obstruction. Postnatal echocardiographic measurements showed that the DA index value was significantly higher in group S (1.5 ± 0.6 vs. 0.8 ± 0.2, *p* < 0.0001) and the cutoff value was 1.25 on the ROC curve (Table 4, Fig. 4).

**Table 4:** DA index after birth compared between groups.

	Group S (n = 13)	Group N (n = 17)	p-value
DA Index	1.5 ± 0.6	0.8 ± 0.2	< 0.0001*

*DA index* distal aortic arch index.

*p < 0.05 between group S and group N.

## Discussion

Multiparametric diagnostic models integrating different ultrasound signs for the detection of CoA in the fetal period were reported in only four studies. Arya et al.^11^ reported the best combination of sensitivity and specificity with a predictive model integrating the angle between the ascending aorta and descending aorta, and between the transverse aorta and descending aorta. Mărginean et al.^12^ reported a combination of RV/LV < 1.5, AoI < 4.2 mm, and AD/AoI > 1.4 gave the overall best predictive accuracy for CoA, although the false-positive rate was 13% and false-negative rate was 44%. Thus, in past reports, multiple measurement sites and formulas have been proposed, and the false-positive and false-negative rates are high.

In the neonatal period and in childhood, Ali et al.^10^ reported that the DA diameter/distance between the second and third branches of the aortic arch was smaller (less than 1.5) in cases of CoA. When we applied this report to our research, we thought that it would be useful because both the false positive and false negative rates were lower than those reported in other studies.

Various developmental aberrations have been proposed as possible explanations for aortic arch abnormalities. Morphological abnormalities proposed as an explanation for CoA concern the fetal blood flow and hemodynamic theory. A reduction in the volume of blood passing through the ascending aorta during the fetal period in CoA, particularly with VSD, that may affect the timing of surgical repair timing, LV outflow obstruction, and tubular hypoplasia of the transverse arch, postnatally leads to the development of CoA. In contrast, Skoda^13^ speculated that the constriction of the aorta is related to closure of the ductus arteriosus extending into the walls of the aorta, a proposal that is referred to as the Skodiac hypothesis or Ductus tissue theory. Yokoyama et al.^14^ determined that mature-phenotype smooth muscle cells in the ductus arteriosus participate in the immediate closure of the ductus arteriosus after birth. The extension of ductus tissue into the aortic wall was followed and led to observations of the CoA phenotype. The molecular mechanism of development of CoA is thought to involve the differential expression of the myosin heavy chain isoforms SM1 and SM2, markers for differentiated smooth muscle cells, which were lower in the intima of the coarctation segments than in that of the ductus arteriosus. These data suggest that smooth cell differentiation is involved in the narrowing aortic isthmus following ductus closure.

Meanwhile, during development of the fetal aortic arch, the left seventh intersegmental artery of the dorsal aorta shortens to form the left subclavian artery after birth. We hypothesized that in addition to the thinning of the distal aortic arch due to decreased blood flow in the ascending aorta, as described in the hemodynamic theory, the left seventh intersegmental artery shortens over a very long distance, and any malfunction during this time would cause the distance between the second and third branches of the aortic arch to become longer. As a result, we speculate that focusing on the second-third branch distance and measuring the DA index will increase the extraction rate of potential patients with CoA.

Our results showed that the DA index is useful for screening neonatal patients to determine whether surgical intervention is needed. A DA index ≧1.28 was useful for detecting CoA in terms of both sensitivity and specificity compared to previous reports. In addition, we examined the DA index using postnatal echocardiography and confirmed that there were significant differences between the two groups, as in previous reports.

To prevent severe postnatal respiratory and circulatory failure, detailed scanning of the fetal heart is critical when a cardiac disproportion is noted, or when cardiac structures are not visualized on routine echocardiographic scanning. The early recognition of CoA reduces morbidity and mortality^15, 16^. As advances in diagnostic and surgical techniques have evolved, early- to mid-term outcomes for patients with CoA are excellent.

This study has several limitations. First, the study design was retrospective with a small number of patients and thus had all the limitations inherent to such a design. Future large multicenter studies sharing the same imaging protocols are needed to develop objective models for risk assessment in fetal patients and to ascertain the actual diagnostic performance of prenatal echocardiography in detecting this anomaly. Secondly, despite innovations in fetal imaging technology, it is possible to visualize the neck vessels of almost all fetuses; in some cases, the difficulty of obtaining this view may be influenced by the position of the fetus and by the obesity of the mother. In these cases, reassessment was needed with re-examination.

In conclusion, the prenatal detection rate of CoA may be improved when the DA index is used. The implementation of this assessment may prevent neonates from experiencing circulatory shock.

## Supplementary Information

Supplementary Information.

## Data Availability

The datasets generated and/or analyzed during the current study are available from the corresponding author upon reasonable request.
