# Supplementary material for: Utility of novel echocardiographic measurements to improve prenatal diagnosis of coarctation of the aorta
Source: Sci Rep. 2023 Mar 25;13:4912. doi: 10.1038/s41598-023-31749-8 (PMC10039932; doi:10.1038/s41598-023-31749-8)
Supplement: Supplementary file 1 — Supplementary Information. [file 41598_2023_31749_MOESM1_ESM.pdf]

Utility of novel echocardiographic measurements to improve prenatal diagnosis of coarctation of the aorta

Takuya Fujisaki[1)], Yoichiro Ishii[1)], Kunihiko Takahashi[1)], Masayoshi Mori[1)], Kumiyo Matsuo[1)], Dai Asada[1)], Hisaaki Aoki[1)], Sanae Tsumura[2)], Shigemitsu Iwai[2)], and Futoshi Kayatani[1)]

1) Department of Pediatric Cardiology, Osaka Women’s and Children’s Hospital, Osaka, Japan
2) Department of Cardiovascular Surgery, Osaka Women’s and Children’s Hospital, Osaka, Japan

Supplementary Fig.S1 The inter-observer variability

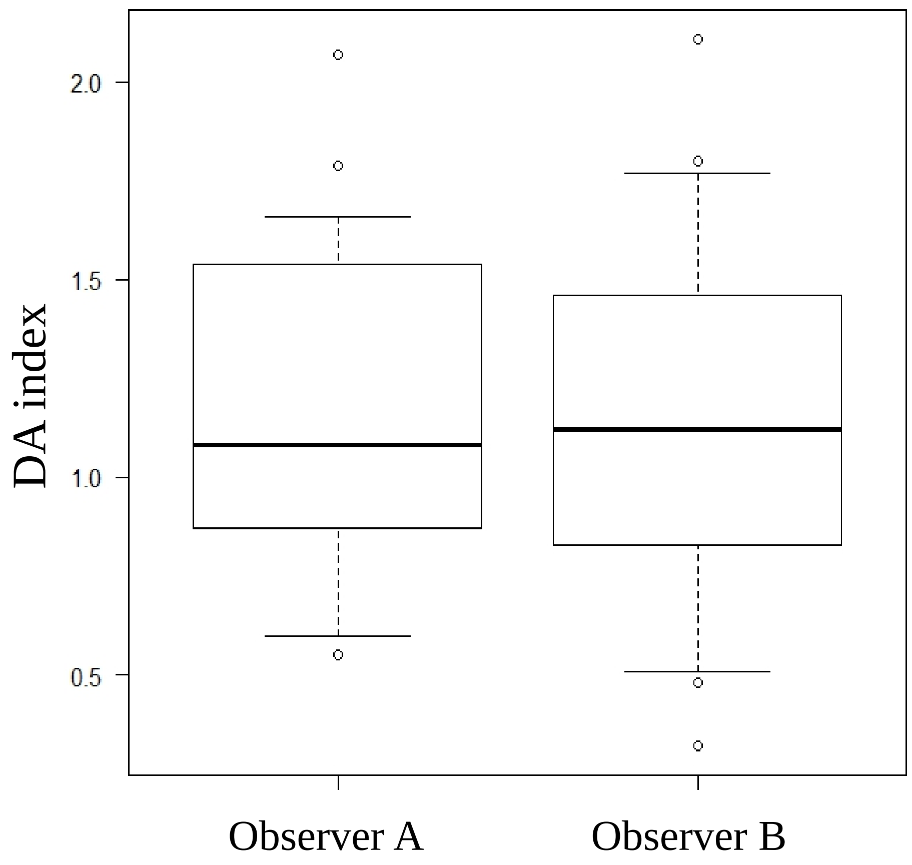

Intraclass correlation coefficients (ICC) was 0.685, which is highly correlated between examiners and there was no inter-rater error.
